# Three Chemically Distinct Floral Ecotypes in *Drakaea livida*, an Orchid Pollinated by Sexual Deception of Thynnine Wasps

**DOI:** 10.3390/plants11030260

**Published:** 2022-01-19

**Authors:** Alyssa M. Weinstein, Björn Bohman, Gavin R. Flematti, Ryan D. Phillips

**Affiliations:** 1 Research School of Biology, Australian National University, Canberra, ACT 2600, Australia; alyssa.weinstein@anu.edu.au (A.M.W.); R.Phillips@latrobe.edu.au (R.D.P.); 2 School of Molecular Sciences, The University of Western Australia, Crawley, WA 6009, Australia; gavin.flematti@uwa.edu.au; 3 Department of Plant Protection Biology, Swedish University of Agricultural Sciences, 23422 Lomma, Sweden; 4 Department of Ecology, Environment and Evolution, La Trobe University, Melbourne, VIC 3086, Australia; 5 Department of Biodiversity, Conservation and Attractions, Kings Park Science, Perth, WA 6005, Australia

**Keywords:** ecotypes, sexual deception, pollinator distribution, floral volatiles, (methylthio)phenols

## Abstract

Sexually deceptive orchids are unusual among plants in that closely related species typically attract different pollinator species using contrasting blends of floral volatiles. Therefore, intraspecific variation in pollinator attraction may also be underpinned by differences in floral volatiles. Here, we tested for the presence of floral ecotypes in the sexually deceptive orchid *Drakaea livida* and investigated if the geographic range of floral ecotypes corresponded to variation in pollinator availability. Pollinator choice trials revealed the presence of three floral ecotypes within *D. livida* that each attracts a different species of thynnine wasp as a pollinator. Surveys of pollinator distribution revealed that the distribution of one of the ecotypes was strongly correlated with that of its pollinator, while another pollinator species was present throughout the range of all three ecotypes, demonstrating that pollinator availability does not always correlate with ecotype distribution. Floral ecotypes differed in chemical volatile composition, with a high degree of separation evident in principal coordinate analysis. Some compounds that differed between ecotypes, including pyrazines and (methylthio)phenols, are known to be electrophysiologically active in thynnine wasp antennae. Based on differences in pollinator response and floral volatile profile, the ecotypes represent distinct entities and should be treated as such in conservation management.

## 1. Introduction

One of the most specialised pollination strategies is that of sexual deception [1,2], in which pollination typically occurs via sexual attraction of male insects to a flower through chemical mimicry of female sex pheromones [3,4,5,6,7]. While sexual deception has been reported in the Asteraceae [8] and Iridaceae [9], it is most prevalent among the Orchidaceae [10,11]. Due to the high specificity of insect sex pheromones, sexually deceptive orchids frequently have only a single pollinator species, with closely related orchids typically exploiting different pollinator species [12,13,14,15,16].

Closely related sexually deceptive orchids often attract pollinators using structurally similar semiochemicals (pheromones or other inter-organism signalling compounds). For example, to attract thynnine wasp pollinators, co-occurring *Chiloglottis* species use different combinations of one or two compounds out of a pool of structurally similar cyclohexanediones (chiloglottones) [6,14]. Similarly, most European *Ophrys* studied to date attract native bees as pollinators using different blends of alkanes and alkenes, with an overlap in compounds between orchid species [7,17,18,19]. However, as more species of sexually deceptive orchids are studied, there is increasing evidence that a diverse range of semiochemical systems can be used within an orchid genus. For example, rather than alkenes and alkanes, one species of *Ophrys* uses carboxylic acids to attract a scoliid wasp pollinator [20]. In the Australian genus *Caladenia*, two species use (methylthio)phenols to attract *Campylothynnus* pollinators [21,22], while another species attracts a member of a different wasp genus, *Zeleboria*, using a monoterpene and an acetophenone [23].

In sexually deceptive orchids, floral odour is thought to play an important role in adaptation to particular pollinators (e.g., [14]), reproductive isolation [24,25] and subsequent speciation. Furthermore, in this group of orchids it is predicted that the first floral trait to diverge during speciation will be the chemical traits associated with pollinator attraction [2,25]. While morphological traits are often critical for pollination in sexually deceptive orchids (e.g., [26]), unlike chemistry, a pronounced shift in morphology is not typically needed to attract a novel pollinator species. Accordingly, sexually deceptive orchids can have chemically different floral ecotypes that are often morphologically extremely similar, or even indistinguishable to the human eye. Indeed, these ecotypes have often first been recognised based on pollinator observations and subsequent choice trials between pollinators, rather than being detected during morphological studies [13,25,27,28,29]. Such morphologically cryptic ecotypes are of interest for understanding the speciation process [25,29,30,31] but could also have important management implications for rare species [28,32].

Chemical composition of floral volatiles has proved an informative trait in distinguishing morphologically cryptic taxa in a number of plant species [33,34,35,36,37,38]. For example, in the sexually deceptive genus *Chiloglottis*, co-occurring morphologically cryptic taxa can be differentiated based on a combination of one or two specific pollinator-attracting compounds [25]. However, this approach requires detailed knowledge of the semiochemicals responsible for pollination attraction. An alternative method of distinguishing between potentially cryptic sexually deceptive orchid taxa is to analyse the entire chemical composition of a flower using multivariate analyses of gas chromatography/mass spectrometry (GC/MS) data from floral extracts [39,40]. The use of such multivariate analyses can provide a high degree of discriminatory power between taxa—as has been successfully demonstrated in *Ophrys*. For example, [40] found that three *Ophrys* taxa of uncertain taxonomic rank, previously distinguished by their attraction of different pollinator species, could be distinguished with 94% accuracy based on floral chemical composition, supporting their status as separate species. Supplementing this data with electroantennographic detection coupled with gas chromatography (GC-EAD) can provide supporting evidence by testing if chemicals that differ between ecotypes can be perceived by pollinators. This technique also represents a useful starting point for determining the compounds responsible for pollinator attraction [6,7,14,17,41,42,43,44].

**Figure 1:**
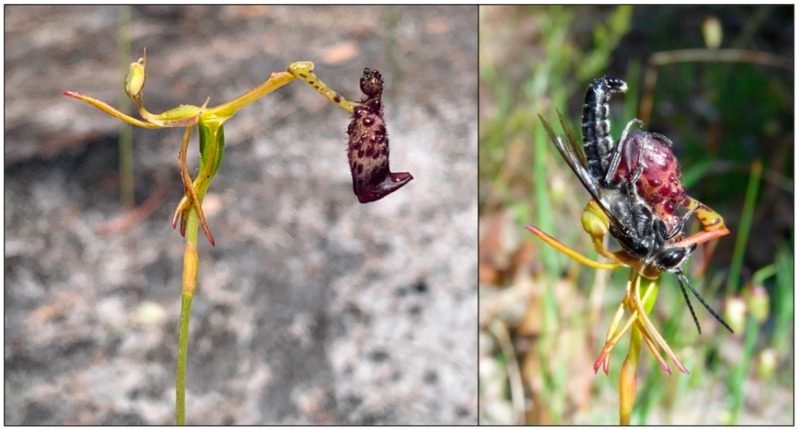
A *Drakaea livida* flower in its natural position (**left**) and with the hinge flipped over by the *Zaspilothynnus nigripes* pollinator (**right**) (photo credits A. Weinstein (**left**) and S. Bond (**right**)).

*Drakaea* is a genus of sexually deceptive orchid endemic to south-western Australia, where all species studied thus far are pollinated by thynnine wasps (Phillips et al., 2014). In *Drakaea glyptodon*, pollinators are attracted with a blend of alkylpyrazines and hydroxymethylpyrazines [43,44], while *Drakaea micrantha* attracts a different pollinator species using hydroxymethylpyrazines in combination with a drakolide, a β-hydroxylactone [45]. Experimental evidence for morphologically cryptic floral ecotypes has been found in three of the ten species of *Drakaea*. In *D. elastica*, a northern and a southern form attract different pollinator species [28]. In *D. concolor*, populations nested within the middle of the species distribution attract a second pollinator species in addition to the primary pollinator attracted by the other populations [29]. The third case of ecotypes appears to be present in *D. livida* (Figure 1), where populations from different geographic areas attract alternative pollinator species [15,43], which respond electrophysiologically to different semiochemicals [42,46].

The thynnine wasp *Zaspilothynnus nigripes* was the first species recorded as a pollinator of *D. livida* [47]. However, more recent studies have confirmed that two additional thynnine species pollinate particular populations of *D. livida*: an undescribed species of *Catocheilus* [42,48] and *Zaspilothynnus dilatatus* [15]. The attraction of *Catocheilus* sp. to *D. livida* is mediated by a blend of an alkylpyrazine and hydroxymethylpyrazines found in the labellum [42,43]. A different hydroxymethylpyrazine found both in flowers that attract male *Z. nigripes* and in sexually calling female *Z. nigripes* was found to be electrophysiologically active to males of this pollinator species [46]. A taxonomic revision of *Drakaea* noted subtle qualitative morphological differences at some populations of *D. livida*, and suggested that further investigation of their taxonomic status was warranted [47].

Given the observation of multiple pollinator species in *D. livida*, we tested for the presence of floral ecotypes, investigated patterns of chemical divergence and pollinator availability across the geographic range of *D. livida*, and determined for the first time which floral compounds were electrophysiologically active to *Z. dilatatus*. It was hypothesised that: (1) *D. livida* is composed of ecotypes, as recognised by differences in pollinator response, (2) the distribution of plants that attract different pollinator species (potential floral ecotypes) correlates with the availability of their pollinator species, (3) the floral volatile composition of plants that attract different pollinator species (potential floral ecotypes) differs, and (4) the presence of electrophysiologically active compounds will vary according to the pollinator species attracted.

## 2. Results

### 2.1. Determining the Pollinator Species of D. livida Populations

**Figure 2:**
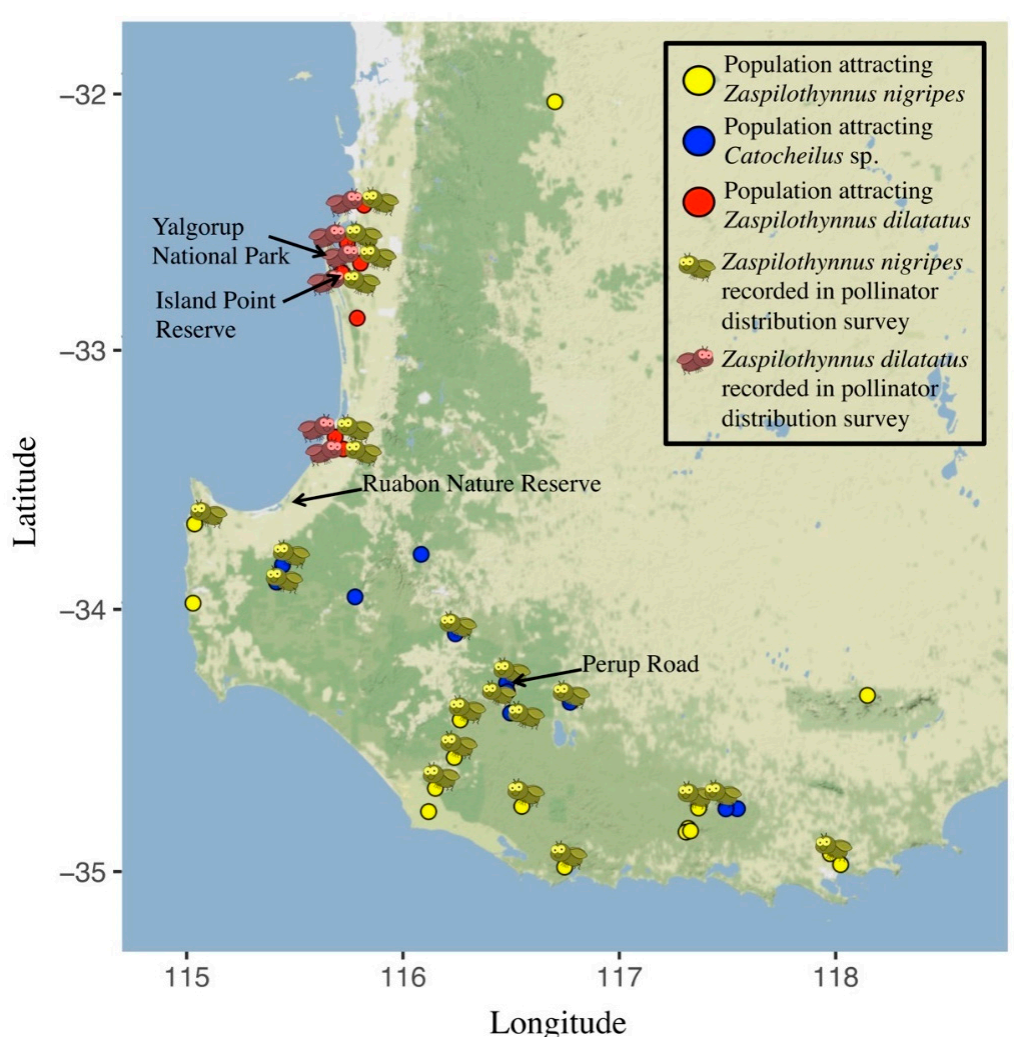
Distribution of populations of *Drakaea livida* that attract *Zaspilothynnus nigripes* (yellow circles), *Catocheilus* sp. (blue circles), and *Zaspilothynnus dilatatus* (red circles) showing which pollinator species were detected in the pollinator survey: *Z. nigripes* present (yellow wasp), *Z. dilatatus* present (red wasp).

Of the 33 populations of *D. livida* tested across its geographic range, wasps were caught to flowers from 28 of these populations. Only previously recorded pollinator species of *D. livida* (*Zaspilothynnus nigripes*, *Z. dilatatus*, *Catocheilus* sp.) were detected. All flowers tested from within a single population attracted the same pollinator species (average 3.93 ± 0.88 SE flowers tested per population). Flowers from fifteen populations were found to attract the pollinator *Z. nigripes*, which displayed copulatory behaviour with the flowers (Figure 2, Supplementary Table S1,). Flowers from five populations were found to attract *Catocheilus* sp. Interestingly, despite *Catocheilus* sp. being a confirmed pollinator of *D. livida* [42,48], no wasps of this species (or others) were observed flipping the hinge of flowers belonging to this putative ecotype. However, they were observed to closely approach (within 5 cm) flowers from five populations. Flowers from seven populations, all on the Swan Coastal Plain, were found to attract *Z. dilatatus* (Figure 2, Supplementary Table S1,). *Zaspilothynnus dilatatus* displayed copulatory behaviour with the flowers and was observed to flip the floral hinge as is required for pollination.

### 2.2. Pollinator Choice Experiments

#### 2.2.1. Response of *Zaspilothynnus dilatatus* to Flowers from Populations That Attract *Z. nigripes* and *Catocheilus* sp.

*Zaspilothynnus dilatatus* was only attracted to flowers from local populations, and ignored flowers from populations that attract alternate pollinator species. In phase one of the experiments, no *Z. dilatatus* approached or landed on flowers from populations that attract *Z. nigripes* or *Catocheilus* sp., demonstrating that these flowers are unattractive to *Z. dilatatus*. In phase two (presentation of local flowers from populations that attract *Z. dilatatus*), *Z. dilatatus* was confirmed as present by its response to known attractive flowers in 24 out of the 29 trials (*N* = 60; 2.07 ± 0.21 wasps per trial). Of the 60 *Z. dilatatus* observed approaching flowers, 45% landed, 42% contacted the column by flipping the hinge, and 30% attempted copulation with the flower.

#### 2.2.2. Response of *Zaspilothynnus nigripes* to Flowers from Populations That Attract *Zaspilothynnus dilatatus*

Within the distribution of *Z. dilatatus*, the co-occurring *Z. nigripes* did not respond to flowers of *D. livida* from populations that attract *Z. dilatatus*. No *Z. nigripes* landed on flowers from populations that attract *Z. dilatatus* in either phase one or two of the 20 trials conducted at Island Point Reserve. In 17 of these trials, *Z. nigripes* was confirmed as present when they landed on the flowers from populations that attract *Z. nigripes*, which were added as a control in phase two (*N* = 81, 4.05 ± 1.15 SE responses per trial). While not the focus of this sequential choice experiment, 36 *Z. dilatatus* were caught to the local flowers expected to attract this species, corroborating the results of the choice trials conducted at Yalgorup National Park.

When trials were conducted at Ruabon Nature Reserve, outside of the known geographic ranges of both *Z. dilatatus* (based on museum records) and the populations of *D. livida* that it is attracted to, 142 *Z. nigripes* (15.78 ± 7.56 SE wasps per trial) responded to flowers from populations that attract *Z. nigripes*, while 116 (5.52 ± 1.14 SE wasps per trial) were attracted to flowers from populations that typically only attract *Z. dilatatus* (Supplementary Figure S1). When landing on flowers from populations that attract *Z. nigripes*, 27.5% of *Z. nigripes* conducted the hinge flip behaviour necessary for pollination. When attracted to flowers from populations that normally attract *Z. dilatatus* only, 0.9% of *Z. nigripes* exhibited the hinge flip behaviour. A *G*-test comparing the behaviour of wasps (in terms of proportion of approach, land, hinge flip) to flowers from populations that attract *Z. dilatatus* with populations that attract *Z. nigripes* revealed a significant difference in wasp behaviour to these two groups of plants (*G* = 44.3 and *p <* 0.001).

#### 2.2.3. Response of *Zaspilothynnus nigripes* to Flowers from Populations That Attract *Catocheilus* sp.

No *Z. nigripes* responded to flowers from populations that attract *Catocheilus* sp. in phase one of the 25 choice trials conducted at a population that attracts *Catocheilus* sp. (Perup Road). In phase two, 81 *Z. nigripes* responded to flowers from populations that attract *Z. nigripes*, confirming their presence at the site. Of these responding *Z. nigripes*, 49.3% flipped the hinge of the flowers that attract *Z. nigripes* (1.60 ± 0.47 SE hinge flips per trial). One *Z. nigripes* flipped the hinge of a flower from a population that attracts *Catocheilus* sp. during phase two of the experiment (simultaneous presentation of flowers from populations that attract *Z. nigripes* and from populations that attract *Catocheilus* sp., 0.04 ± 0.04 SE hinge flips per trial).

When the experiment was repeated at Ruabon Nature Reserve, which is outside of the geographic area where *Catocheilus* sp. are involved in pollination, no *Z. nigripes* responded to the flowers that attract *Catocheilus* sp. in phase one of the 17 choice trials. In phase two, 355 *Z. nigripes* responded to the control flowers from populations that attract *Z. nigripes*, with 24.9 % flipping the hinge (5 ± 0.67 SE hinge flips per trial). In phase two, where flowers from both the populations that attract *Catocheilus* sp. and those that attract *Z. nigripes* were simultaneously presented, one *Z. nigripes* responded and flipped the hinge of a flower from a population that attracts *Catocheilus* sp. (0.06 ± 0.06 SE hinge flips per trial).

### 2.3. Correlation of Plant Distribution and Pollinator Availability

Populations that attract different pollinator species occupied largely discrete geographic regions (Figure 2). However, *Z. nigripes* was recorded at populations across the geographic range of *D. livida*, including populations where local orchids attracted *Z. nigripes*, and populations where local orchids attracted only *Z. dilatatus* or *Catocheilus* sp. (Figure 2). A Wilcoxon rank sum test revealed there to be significantly more *Z. nigripes* recorded at populations known to attract *Z. nigripes* (12.00 ± 2.49 SE wasps per trial) than at populations known to attract *Catocheilus* sp. (4.42 ± 1.58 SE wasps per trial, *p* = 0.029, *W* = 68) and populations known to attract *Z. dilatatus* (3.00 ± 1.11 SE, *p* = 0.021, *W* = 43). There was no significant difference between the number of *Z. nigripes* recorded at populations that attract *Catocheilus* sp. and populations that attract *Z. dilatatus* (*p* = 0.864, *W* = 39.5, Wilcoxon rank sum test). There was no significant difference between the number of *Z. dilatatus* and *Z. nigripes* recorded at populations that attract *Z. dilatatus* (*p* = 0.895, *W* = 26, Wilcoxon rank sum test). *Zaspilothynnus dilatatus* was recorded at six out of the seven (85.7 %) populations known to attract *Z. dilatatus*, and was not recorded at any populations known to attract *Z. nigripes* or *Catocheilus* sp. (Table 1). While observed in other experiments, no *Catocheilus* sp. were observed during the pollinator distribution survey.

### 2.4. Floral Volatile Composition of Plants That Attract Different Pollinator Species

**Figure 3:**
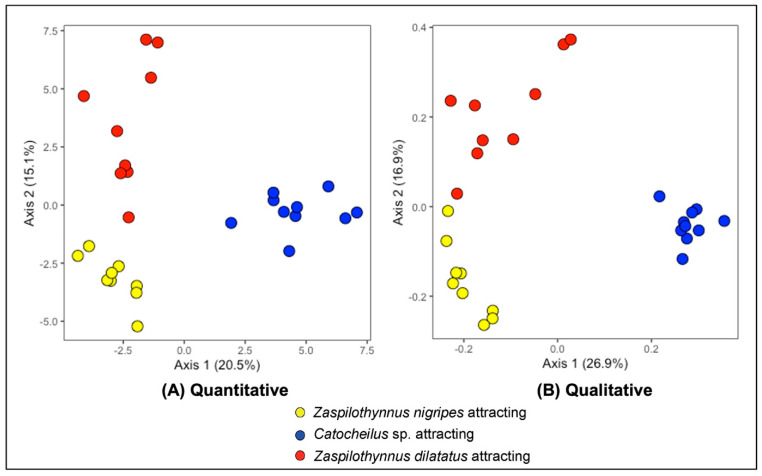
Principal coordinate analyses (PCoA) based on the (**A**) quantitative and (**B**) presence-absence data from 66 compounds detected in the *Drakaea livida* extracts (flowers that attracted *Zaspilothynnus nigripes* = yellow, flowers from populations attracting *Catocheilus* sp. = blue, flowers that attracted *Zaspilothynnus dilatatus* = red). The relative corrected eigenvalues denoting the percentage contribution of each axis to the total variation is displayed in the axes titles.

Flowers that attracted different pollinator species were found to possess different floral volatile compositions. Using floral extracts from specimens with a confirmed pollinator response, 66 compounds met the criteria for inclusion in the multivariate extract analysis. Principal coordinate analyses (PCoA), both quantitative and qualitative, showed three distinct clusters each comprised of samples from populations that attract a single pollinator species (Figure 3 for Axes 1 and 2, Supplementary Figure S2 for Axes 2 and 3). For the quantitative plot, the first three axes contribute 48.5% of the total variation (Axis 1: 20.5%, Axis 2: 15.1%, Axis 3: 12.9%). There was a significant global difference between flowers that attract different pollinator species (PERMANOVA, R^2^ = 0.32, *p* = 0.001). Pairwise comparisons revealed significant differences between all groups of flowers as defined by pollinator response (*Z. nigripes* vs. *Catocheilus* sp. attracting flowers, R^2^ = 0.29, *p* < 0.001; *Z. nigripes* vs. *Z. dilatatus* attracting flowers, R^2^ = 0.22, *p* < 0.001; *Catocheilus* sp. vs. *Z. dilatatus* attracting flowers, R^2^ = 0.26, *p* < 0.001). For the qualitative plot, the first three axes contribute 52.5% of the total variation (Axis 1: 26.9%, Axis 2: 16.9%, Axis 3: 8.7%). There was a significant global difference between flowers that attract different pollinator species (PERMANOVA, R^2^ = 0.48, *p* = 0.001). Pairwise comparisons revealed significant differences between all groups of flowers as defined by pollinator response (*Z. nigripes* vs. *Catocheilus* sp. attracting flowers, R^2^ = 0.46, *p* < 0.001; *Z. nigripes* vs. *Z. dilatatus* attracting flowers, R^2^ = 0.31, *p* < 0.001; *Catocheilus* sp. vs. *Z. dilatatus* attracting flowers, R^2^ = 0.44, *p* < 0.001).

### 2.5. Presence of Electrophysiologically Active Compounds

#### 2.5.1. Gas Chromatography/Mass Spectrometry-Electroantennographic Detection Studies of *Z. dilatatus*

In addition to the six compounds previously reported to be electrophysiologically active in *D. livida* (**1**, **8**–**9**, **12**–**14**, Table 2) to *Z. nigripes* and *Catocheilus* sp. [42,46], analysis of GC/MS-EAD data revealed electroantennographic responses of *Z. dilatatus* to two compounds present in floral extracts from populations that attract this species (Supplementary Figure S3). These compounds were identified by comparisons of retention data and mass spectra, and confirmed by co-injection to be 2-(methylthio)benzene-1,4-diol (**18**) and 4-hydroxy-3-(methylthio)benzaldehyde (**19**), which were both available from a previous study (Bohman et al., 2017) (Table 2).

#### 2.5.2. Screening of Floral Extracts for Electrophysiologically Active Compounds

Of the 347 floral extracts, 292 (84.1%) contained one or more of the electrophysiologically active compounds. Compounds electrophysiologically active to a specific pollinator species were only present in plants attracting that pollinator species. Compound **1** (known to be electrophysiologically active to *Z. nigripes* [46]) was found in 78 flowers, all of which came from populations that attracted *Z. nigripes* (total 111 flowers). Compounds **8–9** and **12–14** (known to be electrophysiologically active to *Catocheilus* sp. [42]) were found exclusively in populations known to attract *Catocheilus* sp. Compound **14** was found in all (113) flowers from populations known to attract *Catocheilus* sp. Compounds **18** and **19** (electrophysiologically active to *Z. dilatatus* in the present study) were found exclusively in populations that attract *Z. dilatatus*. While compound **18** was not detected in the automated extract analyses due to co-elution with **19**, manual screening enabled its detection. Compounds **18** and **19** were found in 46% (57) and 80% (98) of the flowers from populations known to attract *Z. dilatatus* (123). Each of the 28 populations with replicate individuals (mean samples per population = 7.94 ± 1.6 SE) was composed of flowers containing compounds electrophysiologically active to a single pollinator species only.

### 2.6. Compounds That Differ between Ecotypes

A total of 19 compounds that differed between putative ecotypes were identified in the floral extract analyses, seven of which were already known from electroantennographic detection analyses. Of the 12 candidate informative compounds detected only by floral solvent extract analyses, three were identified, one was tentatively identified based on NIST library matches, and eight remain unknown. Compound **20** was identified by co-injection of a synthetic standard as 4-(hydroxymethyl)-2-(methylthio)phenol. Compound **4** was identified by co-injection as 4-(2-hydroxyethyl)-2-methoxyphenol (homovanillyl alcohol). Compound **7** was identified as 3,5,6-trimethylpyrazine-2-carbaldehyde, which may in fact be an artefact from the analysis, formed in the GC-inlet by oxidation [49] and was therefore not included in any analyses. Compound **6** was tentatively identified as a C21-alkene (double bond position not confirmed). The mass spectra and retention indices of the unidentified compounds **2–3**, **5**, **10–11**, and **15–17** did not match any of those in the NIST database or our custom in-house library of mass spectra. All of compounds **1–20** could be reliably detected in extracts using their characteristic mass fragments and RIs presented in Table 2. Full mass spectra of **1**-**20** are presented in Supplementary Figure S4. The suite of informative compounds present in a population remained constant across years (mean number of years sampled per population = 2.7 ± 0.3 SE).

## 3. Discussion

### 3.1. Presence of Floral Ecotypes in Drakaea livida

In accordance with the hypothesis that *D. livida* is comprised of floral ecotypes, the results of pollinator choice trials indicate that three distinct ecotypes are present in *D. livida*, each defined by different pollinator responses. One ecotype is visited exclusively by *Z. nigripes* across its broad geographic range (Ecotype One, Figure 2). Another ecotype (Ecotype Two) consists of populations known to attract *Catocheilus* sp. but not *Z. dilatatus* (Ecotype Two). While flowers from these populations elicited rare responses from *Z. nigripes* (in this study, less than 0.01% of *Z. nigripes* responded to Ecotype Two flowers when they were encountered), the behaviour of *Z. nigripes* when responding to Ecotype Two flowers differed markedly to the behaviour of *Z. nigripes* responding to Ecotype One flowers. When responding to Ecotype Two flowers, a much smaller proportion of *Z. nigripes* flipped the hinge (average of five hinge flips per trial to flowers that attract *Z. nigripes*, vs. 0.06 hinge flips per trial to flowers that attract *Catocheilus* sp.). A third ecotype, which exclusively employs *Z. dilatatus* as a pollinator (Ecotype Three), was found only on the Swan Coastal Plain. *Zaspilothynnus dilatatus* displayed a similar rate of column contact to *Z. nigripes* (42% and 43%, [50]). In summary, there were clear quantitative differences in the species and/or behaviour of pollinator attracted to each ecotype, allowing for the recognition of three ecotypes of *D. livida* based on pollinator response.

While Ecotype Two is clearly supported as a different entity to Ecotypes One and Three based on the behaviour of the wasp species, aspects of the pollination of this ecotype are not yet fully resolved. While *Catocheilus* sp. has been observed to conduct the behaviour necessary for pollination [42,48], given its propensity to approach flowers without landing or flipping the hinge (behaviour that results in a low pollination efficiency) it is plausible that Ecotype Two has an additional undetected pollinator species that contributes to fruit set. If additional pollinator species are present, they are likely to occur in low abundance, or potentially differ in life history to the pollinator species successfully detected with the baiting methodology. Already, there is evidence that the disjunct northernmost population of this ecotype attracts a different, yet closely related species of *Catocheilus* [15], potentially indicating undetected variation within this ecotype. As such, it is possible that populations of *D. livida* at the margins of its range not included in this study may potentially attract different pollinator species and represent additional ecotypic diversity.

While *Z. dilatatus* was the sole pollinator species of Ecotype Three, despite the presence of *Z. nigripes* at sites where Ecotype Three grows, when Ecotype Three was tested outside the geographic range of *Z. dilatatus*/Ecotype Three, infrequent responses of *Z. nigripes* were recorded. These occasional responses could potentially arise due to the greater abundance of *Z. nigripes* at these sites. Outside the range of Ecotype Three, an average of 15.78 *Z. nigripes* were recorded per three-minute trial, while inside the range of Ecotype Three only 4.05 *Z. nigripes* were recorded per three-minute trial. When male thynnine wasps are in greater abundance, and potentially experiencing a higher operational sex ratio when mate searching [51,52], they may be more likely to respond to a broader range of mate signals [53]. Alternatively, within the geographic range of *Z. dilatatus* there may be selection pressure for *Z. nigripes* to recognise and avoid the female sex pheromone of *Z. dilatatus*, while this may not be the case outside of the range of *Z. dilatatus*.

### 3.2. Correlation of Ecotype Distribution and Pollinator Availability

Based on the populations sampled, each ecotype appeared to occupy a largely distinct geographic area: Ecotype One was predominantly found in coastal areas south of the Swan Coastal Plain and on the dry margins of the Jarrah forest, Ecotype Two in inland areas of the southern Jarrah forest, and Ecotype Three on the Swan Coastal Plain (Figure 2). However, the hypothesis that the distribution of the ecotypes correlates with the availability of their pollinator species was not supported for all species. The pollinator survey revealed the distribution of the Ecotype One pollinator *Z. nigripes* to be much broader than that of the Ecotype One orchids (Figure 2), suggesting a potential role for abiotic factors in limiting the geographic range of Ecotype One. The ability to infer patterns of availability are limited in Ecotype Two, given its *Catocheilus* sp. pollinator shows less attraction to flowers than the pollinators of Ecotypes One and Three, and that the pollinator distribution could not be quantified. The pollinator survey revealed the distribution of Ecotype Three to be strongly correlated with the distribution of its pollinator, the Swan Coastal Plain endemic *Z. dilatatus*. However, despite this correlation, the pollinator of Ecotype One (*Z. nigripes*) was present throughout the distribution of Ecotypes Two and Three. The presence of the Ecotype One pollinator throughout the distributions of all three Ecotypes (two of which do not attract it) is in contrast to plant species where floral ecotypes exploit the locally available pollinator species [54,55]. In *Drakaea*, a similar scenario was found in the ecotypes of *D. concolor* [29], where the distribution of the ecotypes did not correlate closely with the abundance of the their respective pollinators.

### 3.3. Floral Volatile Composition of the Ecotypes

The hypothesis that the floral volatile composition of the ecotypes differs was supported. In the multivariate analysis of floral volatile composition, three significantly different clusters were found, each correlating with the attraction of a different specific pollinator. The hypothesis that the presence of electrophysiologically active compounds would vary according to the pollinator species attracted was also supported. In addition to the pyrazines known to be electrophysiologically active to Ecotypes One and Two, respectively [42,46], in the present study, two (methylthio)phenol compounds were found to be electrophysiologically active to the Ecotype Three pollinator *Z. dilatatus*. All electrophysiologically active compounds were found exclusively in flowers of the ecotype pollinated by the pollinator they were perceived by. This contrasts with some other sexually deceptive pollination systems where there is overlap in some of the electrophysiologically active compounds between related species, for both orchid and pollinator [14,17,20,56,57].

It is interesting to note that, despite the large number of flowers sampled, no extracts displayed evidence of an intermediate phenotype containing a mix of electrophysiologically active compounds normally associated with different pollinator species. Further, the combined pollinator and chemical data suggested that only one ecotype occurred per population of *D. livida*. Nonetheless, mixed phenotype individuals may yet occur at a low abundance in some populations of *D. livida*, potentially where the ranges of the ecotypes adjoin. Despite this possibility, the ecotype specificity of the electrophysiologically active compounds, taken together with the attraction of different pollinator species, is suggestive of a level of reproductive isolation between ecotypes.

To test whether the electrophysiologically active compounds are responsible for the attraction of the different pollinator species, field trials should be undertaken. It is possible that the compounds responsible for pollinator attraction include compounds not detected via GC-EAD or by our compound screening process [22,58]. For example, compounds that are important for pollinator attraction, but are shared between the three ecotypes, would not be identified through our screening for qualitative differences in compounds between ecotypes.

### 3.4. The Discovery of (Methylthio)Phenols in D. livida

The discovery of (methylthio)phenols in *D. livida* adds a new class of pollinator-perceived compounds known to occur in *Drakaea*, in addition to the previously reported pyrazines and drakolide (a β-hydroxylactone [42,43,44,45,46]. Remarkably, within *D. livida* these structurally diverse compounds ((methylthio)phenols and pyrazines) occur in different ecotypes—an unexpected situation for plant populations that are ostensibly each other’s closest relatives. While there is a precedent for the use of structurally diverse compounds within a genus of sexually deceptive orchid [21,24,46], it is remarkable that within a single species, two different wasp genera are attracted, which respond to compounds with very different structures. The production of these structurally distinct compounds is expected to occur through different biosynthetic pathways that are associated with different suites of by-products and intermediates. This could explain the high degree of chemical differentiation between the ecotypes in the principal co-ordinate analysis, which likely included some compounds not directly involved in pollinator attraction. The potential use of different biosynthetic pathways in different ecotypes is not the expected scenario for closely related taxa, and hints at an interesting evolutionary origin.

The discovery that (methylthio)phenols are present in Ecotype Three and are perceived by the pollinator *Z. dilatatus* presents an interesting case of convergent evolution of floral volatiles. In two species of *Caladenia*, the same two (methylthio)phenols (**18** and **19**) perceived by *Z. dilatatus* underlie the attraction of sexually deceived pollinators in the thynnine wasp genus *Campylothynnus* (in one case as part of a blend with **20** and an additional (methylthio)phenol compound) [21,22]. While the sharing of a pyrazine between *Drakaea* and *Caladenia* has been previously reported [59], this example in *D. livida* represents the first case where the shared compound(s) are known to be perceived by pollinators in both orchid genera. These (methylthio)phenol compounds are not currently known as semiochemicals in any other organisms, yet given that they are perceived by two different genera of thynnine wasps, they may represent an important class of semiochemicals within the clade that contains *Zaspilothynnus* and *Campylothynnus* and many other orchid-pollinating wasps (see [15]).

It is of interest that the (methylthio)phenols and all but one of the compounds found to be electrophysiologically active in *D. livida* [42,46] were also detected in the screening of extracts for compounds that differed between ecotypes. The sole exception was compound **18**, which co-eluted with compound **19** (both electrophysiologically active) in the floral extract analyses. While not the focus of the present study, our results suggest that the screening of floral extracts for compounds whose presence correlates with the attraction of a specific pollinator species may prove an effective complementary method to electrophysiology for finding candidate pollinator attractant compounds. This approach may be particularly useful in systems where pollinator availability is limited. Due to differing detection limits under different analytical conditions (e.g., differing columns) and the inherent reliance of EAD on variably responsive antennae [60], multiple opportunities exist for semiochemicals to be missed in a given analysis. As such, in many study systems methods should ideally be used to complement one another and not in isolation.

### 3.5. Conservation Implications of the Presence of Ecotypes

The present study found strong evidence for three chemically distinct floral ecotypes of *D. livida* that each occupy a different geographic region. These ecotypes may represent different evolutionary lineages and potentially could prove to be discrete taxa under the biological species concept [61]. Detailed studies in areas where the ecotypes occur in close proximity are now needed to test for gene flow and ongoing reproductive isolation between ecotypes. Nonetheless, differences in pollinator attraction and differences in the associated floral chemistry are strongly suggestive of local adaptation. As such, it is recommended that, until evidence is presented to the contrary, the three ecotypes be treated as distinct entities in conservation management.

Conservation concerns may stand for Ecotype Three, which thus far is only known from nine remnant bushland reserves on the Swan Coastal Plain, where it grows in *Kunzea ericifolia* thickets among mixed *Eucalyptus* and *Banksia* woodland. The Swan Coastal Plain is a known hotspot for orchid rarity, where regional endemics have become rare through extensive habitat clearing for agriculture and urban development [62,63,64]. As such, it is likely that Ecotype Three may be rare and threatened by habitat loss. It is recommended that further research be conducted to determine the geographic extent of the ecotypes. A critical component of such an investigation will be determining reliable method/s of identifying the ecotypes—baiting for pollinators is not ideal as it requires the picking of fresh flowers. Multivariate morphological analyses focusing on populations known to be different ecotypes may uncover undiscovered morphological differences that could assist with identification in the field. To this end, our chemistry analysis described here provides a useful way of distinguishing the *D. livida* ecotypes and could help inform future morphological studies.

## 4. Materials and Methods

### 4.1. Study Species

In *Drakaea,* pollinators are attracted primarily by chemical mimicry of flightless female thynnine wasps [44], though a level of visual mimicry may be important once the pollinator arrives at the flower [65]. Sexually excited male wasps attempt to pick up and fly off with the odour-producing labellum [50]. Due to the presence of the unusual hinged labellum in *Drakaea* (Figure 1), in attempting to fly off and copulate with the orchid labellum, the momentum of the wasp causes the floral hinge to swing the wasp upside down, bringing its thorax into contact with the column, where the pollinia and stigma are housed [5]. This flipping of the hinge by the wasp is required for pollination to occur. *Drakaea* plants do not flower every flowering season (spring), and when they do they produce only a single scape bearing a single flower [47]. All *Drakaea* species are reliant on the mycorrhizal fungus *Tuslasnella secunda* for germination and annual growth [48,66]. *Drakaea livida* is almost entirely restricted to well-drained, grey sandy soils [47]. The species is one of the more geographically widespread species of *Drakaea* and occurs in a range of vegetation communities [47].

### 4.2. Testing for the Presence of Floral Ecotypes in D. livida

In testing the hypothesis that *D. livida* is comprised of floral ecotypes, two experiments were implemented using flowers to bait for pollinators. Baiting for pollinators entails the artificial presentation of picked flowers in natural habitat, which in systems involving sexual deception of thynnine wasps typically leads to the attraction of the pollinator species within minutes if they are present [5,67]. In the present study, to achieve new pollinator responses, flowers were relocated a minimum of 10 m following each three-minute presentation. The first experiment comprised a survey to determine which wasp species pollinate different populations of *D. livida* across its geographic range. Using the outcome of the first experiment, pollinator choice experiments were then conducted to determine the response of different pollinator species to different populations of orchids. Baiting was conducted on sunny days ≥20 °C when thynnine wasps are most active [5]. Flowers were kept at 4 °C in a portable refrigerator between baiting experiments. Experiments were conducted during September and October to coincide with the flight period of the pollinator species.

### 4.3. Determining the Pollinator Species of D. livida Populations

To determine which species of wasp are attracted to *D. livida* flowers across its distribution, flowers from 33 populations across the geographic range of *D. livida* were individually ‘baited with’ (for populations and samples sizes see Supplementary Table S1). Flowers were baited with in areas of natural habitat within the range of *D. livida* that were either in the vicinity of *D. livida* populations or in areas where pollinator species were known to be abundant. Wasps observed flipping the hinge of the flower (as required for pollination) were caught in an insect net for identification. Where possible, wasps were captured in cases where they closely approached flowers, but did not land. Voucher specimens of *D. livida* have been deposited in the West Australian Herbarium (voucher numbers in Supplementary Table S2). Locations of the populations and pollinator species attracted were mapped, with the addition of a population that attracts *Catocheilus* sp. reported previously [48], for which no pollination data were collected during the present survey.

### 4.4. Pollinator Choice Experiments

If ecotypes are present in *D. livida*, we would expect populations of pollinators to show differing responses to plants of *D. livida* depending on which population they were collected from and what their natural pollinator species is. To test for this possibility, we conducted pollinator choice experiments consisting of a series of sequential trials based on the methodology of Bower [68]. Each trial was conducted at one location and consisted of two sequential phases. In the first phase, a foreign flower is presented alone to test if the local pollinator species responds to the foreign flower. In the second phase, a local flower is presented alongside the foreign flower to confirm the presence of the local pollinator species. While not being presented, bait flowers were kept in an airtight cooler box. Choice trials could not be conducted for *Catocheilus* sp. due to its infrequent response to flowers compared to other *Drakaea* pollinators. *Catocheilus* sp. visit flowers at a very low frequency, and when they do, the behaviour necessary for pollination is infrequently displayed (R. Phillips personal observation, corroborated in the results of the present study). Due to differences in pollinator abundance and behaviour between study sites, the methodology of the experiments varies slightly, so is explicitly stated below.

#### 4.4.1. Response of *Zaspilothynnus dilatatus* to Flowers from Populations That Attract Other Pollinator Species

To test whether *Z. dilatatus* was attracted to flowers from populations that attract *Z. nigripes* and *Catocheilus* sp., 29 sequential two-phase choice trials [68] were conducted at a site in Yalgorup National Park (−32°41′21.8″ S 115°38′17.7″ E) where *Z. dilatatus* is common and *Z. nigripes* does not occur. In phase one, a flower from a population that attracts *Z. nigripes* (11 flowers from four populations) and a flower from a population that attracts *Catocheilus* sp. (10 flowers from one population) were presented for five minutes while flowers from populations that attract *Z. dilatatus* were kept in an airtight cooler box. In phase two, a flower from a population that attracts *Z. dilatatus* (nine flowers individually presented from four populations) was randomly selected to confirm the presence of *Z. dilatatus* and was presented at a minimum distance of one meter from the phase one flowers. For each responding wasp, four categories of responses were scored, modified from Peakall [67]: (1) approach only to the flower (within 30 cm), (2) landing on the flower with an absence of hinge flipping or copulation, (3) landing on the flower and subsequently flipping the hinge, and (4) attempting copulation with the flower.

#### 4.4.2. Response of *Zaspilothynnus nigripes* to Flowers from Populations That Attract *Zaspilothynnus dilatatus*

To test whether *Z. nigripes* is attracted to flowers from populations that attract *Z. dilatatus*, 20 sequential choice trials were conducted at Island Point Reserve (−32°45′26.39″, 115°41′24.10″), a site in the middle of the geographic range of the populations that attract *Z. dilatatus*, and where *Z. nigripes* is abundant [50]. In phase one, individual flowers from populations that attract *Z. dilatatus* were presented alone for a three-minute period. In phase two (three-minute presentation), a flower from a population that attracts *Z. nigripes* was presented alongside a flower from a population that attracts *Z. dilatatus* as a control to confirm the presence of *Z. nigripes*. As *Z. dilatatus* also occurs at Island Point Reserve and is indistinguishable from *Z. nigripes* in flight, only wasps that landed on flowers were recorded, as these could be caught and identified in the field using differences in the colour of the legs and the shape of the clypeus.

To determine the response of *Z. nigripes* to flowers from populations that attract *Z. dilatatus*, but outside the distribution of *Z. dilatatus* (based on museum records of *Z. dilatatus*), choice trials presenting flowers from populations that attract *Z. dilatatus* were conducted at Ruabon Nature Reserve, where *Z. nigripes* is abundant [69]. A flower from populations that attract each pollinator species (flowers from populations that attract *Z. dilatatus*: total of eight flowers from four populations, flowers from populations that attract *Z. nigripes*: total of four flowers from three populations) was presented alternately in three-minute trials. Wasp behaviour was scored according to the three mutually exclusive categories ‘approach only’, ‘land’, and ‘land with hinge flip’. A *G*-test was conducted in Genalex [70,71] to compare the response categories of *Z. nigripes* to the populations that attract the two different pollinator species.

#### 4.4.3. Response of *Zaspilothynnus nigripes* to Flowers from Populations That Attract *Catocheilus* sp.

To test whether *Z. nigripes* pollinates flowers from populations that attract *Catocheilus* sp., 25 sequential choice trials were conducted at Perup Road (−34°18′0.54″, 116°25′58.02″). This site in the middle of the geographic range of populations that attract *Catocheilus* sp. and *Z. nigripes* was known to be abundant there. In phase one of each choice trail (three minutes), a flower from a population that attracts *Catocheilus* sp. (total of four flowers from two populations) was presented alone. In phase two (three minutes), a flower from a population that attracts *Z. nigripes* (six flowers from six populations presented) was presented alongside the flower from a population that attracts *Catocheilus* sp. as a control to confirm the presence of *Z. nigripes*.

To determine the response of *Z. nigripes* to flowers from populations that attract *Catocheilus* sp. outside the currently known distribution of the ecotype, 17 sequential choice trials were conducted at Ruabon Nature Reserve. In phase one, a flower from populations that attracts *Catocheilus* sp. was presented alone, and in phase two flowers from populations that attract *Z. nigripes* were removed from a sealed container and presented alongside the flowers from populations that attract *Catocheilus* sp. as a control to confirm the presence of *Z. nigripes*.

### 4.5. Correlation of Plant Geographic Range and Pollinator Availability

To test whether the distribution of plants that attract different pollinator species correlated with the availability of their pollinator species, we surveyed for pollinators of *D. livida* across its geographic range. In total, 28 different populations of *D. livida* were surveyed for pollinator abundance between 2015 and 2017. Nine populations that had attracted *Z. nigripes* either in the baiting survey or in earlier studies (e.g., [48,50]), and seven populations that had attracted *Z. dilatatus* either in the baiting survey or in earlier studies (e.g., [15,69])*,* were included. Due to the infrequent response of *Catocheilus* sp., in addition to including populations that attracted *Catocheilus* sp. in the baiting survey, an additional eight populations containing the known *Catocheilus* sp. attractants (3,5,6-trimethylpyrazin-2-yl)methyl-3-methylbutanoate and 2-(3-methylbutyl)-3,5,6-trimethylpyrazine [43] were included. To quantify the availability of *D. livida* pollinator species, at each population six two-minute baiting trials were conducted and the number and species of wasp landing on bait orchids were recorded [48]. Using this method, presence/absence survey results have been shown to be 90 % repeatable between years [48]. Due to the slightly earlier flowering period of *D. livida* populations from the northern end of the geographic range, only populations that attract *Z. dilatatus* and *Z. nigripes* were presented at the early-flowering Swan Coastal Plain populations. Flowers from populations that attract each of the three pollinators were presented at the later flowering southern populations. To enable the comparison of the availability of different pollinator species, differences in the number of responding wasps detected at populations that attract different pollinators were tested using a Wilcoxon rank sum test in Rv3.5.1 [72].

### 4.6. Floral Volatile Composition of Plants That Attract Different Pollinator Species

To test the hypothesis that plants that attract different pollinator species also differ in their floral volatile composition, we conducted multivariate analyses of GC/MS data from floral extracts of flowers from populations that attract different pollinator species. For all extractions, individual labella were extracted for 24 h at room temperature in 100 μL of dichloromethane containing 100 ng tert-butyl benzene (Sigma-Aldrich, 99.8%) as an internal standard, after which period the extracts were stored at −20 °C until analysis.

For populations that attract *Z. nigripes* and *Z. dilatatus,* picked flowers were used as baits to confirm the attractiveness of flowers to their pollinator species before extraction. To ensure that extracts were made from fresh flowers, flowers were presented to pollinators within an hour of collection and were used as baits for a maximum period of one hour prior to extraction. Replicate flowers that attract the same pollinator species (predicted from pollinator survey) were sampled over multiple days to address any temporal effect of the sampling conditions, e.g., the effect of sunlight [73]. For all floral extracts, only the labellum was used, as previous dissection experiments have shown that the labellum is the source of the pollinator attractants in *D. livida* [50]. Three populations that are attractive to *Z. nigripes* and three populations that are attractive to *Z. dilatatus* were sampled on different days to give a total of ten fresh flowers collected per pollinator species across the season. For these populations, as soon as a pollinator landed on a flower, it was caught for subsequent identification and the flower was immediately extracted. Only flowers to which pollinators responded were extracted.

For populations that attract *Catocheilus* sp., where pollinator responses are rare, ten flowers were sampled from the Frosty Road population, which was used in the chemical studies that previously identified tetrasubstituted pyrazines as the key compounds that underlie the attraction of *Catocheilus* sp. [42,43]. While the responses by *Catocheilus* sp. in floral baiting trials were infrequent (see results), prior to extraction Frosty Road flowers were presented to any potential pollinators for a matching period of time (1 h) to flowers from other populations to ensure comparable treatment of flowers.

GC/MS analyses of the floral extracts were conducted using an Agilent 5973 Network Mass Selective Detector connected to an Agilent 6890N Network GC system equipped with an HP5MS-UI column (30 m × 0.25 mm × 0.25 μm film thickness, Agilent), using helium as a carrier gas at 1 mL/min. Peak detection, deconvolution, and quantification was conducted using the EasyGC python pipeline (https://libraries.io/github/dkainer/easyGC, accessed on 13 January 2022), based on PyMS python library [74]) with the default parameters. Compounds that occurred in less than three of the thirty flowers were excluded from the analyses.

To test for differences in chemical composition between plants that attract different pollinators, we analysed our data both quantitatively and qualitatively. We took this dual analytical approach as a quantitative analysis is needed to distinguish between ecotypes that differ in the ratio of compounds only, but qualitative analysis may be less affected by varying sampling and analysis conditions. As the GC/MS data contained zero values, data were fourth-root transformed, centred, and scaled prior to quantitative analysis [75,76]. For the qualitative analysis, data were presence-absence transformed, with all values greater than zero being set to one, prior to analyses. For both analyses, to visualise the difference in qualitative chemical composition between samples, a Jaccard distance matrix [75,77] was calculated using the package ‘vegan’ [78], from which a principal coordinate analysis (PCoA) was conducted using the package ‘ape’ [79] in R v 3.5.1 [72]. To test for differences between groups of flowers that attract different pollinator species, Permutational Multivariate Analysis of Variance (PERMANOVA) was conducted using the vegan ‘adonis’ function. Pairwise comparisons between groups were calculated for 100,000 permutations using a Holm correction for multiple comparisons in the R package ‘funfuns’ (https://github.com/Jtrachsel/funfuns, accessed on 6 January 2022).

### 4.7. Presence of Electrophysiologically Active Compounds

Previous studies have already detected compounds in *D. livida* that are electrophysiologically active in *Z. nigripes* [46] and *Catocheilus* sp. [42]. To complement this data, gas chromatography/mass spectrometry-electroantennographic detection (GC/MS-EAD) was conducted for the third pollinator species (*Z. dilatatus*) using floral extracts from populations that attract this pollinator species. To test if the presence of electrophysiologically active compounds varied according to the pollinator species attracted, extracts from populations across the range of *D. livida* with known pollinator species were screened for the presence of all known electrophysiologically active compounds in *D. livida* either detected in the present or previous studies [42,46].

#### 4.7.1. Gas Chromatography/Mass Spectrometry-Electroantennographic Detection Studies of *Z. dilatatus*

Male *Z. dilatatus* were caught with insect nets to flowers from populations of *D. livida* known to attract this species, and kept at 4 °C until use in GC/MS-EAD experiments. Single antennae were tested against pooled single labella extracts from populations that attract *Z. dilatatus* that were concentrated under a nitrogen stream, and synthetic compounds (prepared as per [21]). GC/MS-EAD data were recorded using a HP GCD 1800A equipped with a BPX5 column ((5% phenyl dimethylpolysiloxane), 30 m × 0.25 mm × 0.25 μm film thickness, SGE Australia), using helium as a carrier gas. A GC effluent splitter was used to split the flow to the MS and EAD. The split for EAD was passed through a Syntech effluent conditioner (Syntech, Kirchzarten, Germany) containing a heated transfer line, with the outlet placed in a purified and humidified airstream, where the stainless steel electrodes holding the antenna were contained in a glass tube. For each EAD run, an excised antenna with the tip cut off was mounted on the holder (consisting of two electrodes) using electrode gel. The electrodes were connected to a PC via a Syntech Intelligent Data Acquisition Controller (IDAC2) for the recording of EAD signals in the Syntech software package GC-EAD/2014 (freely available from http://gcead.sourceforge.net/download.html, accessed on 13 January 2022). For all observed EAD responses, linear retention indices were calculated to enable the comparison of data across instruments and experimental conditions. Retention indices and mass spectra of compounds that elicited an electrophysiological response were compared to those of matches returned by searching: (i) the National Institute of Standards and Technology (NIST) database (NIST-17), and (ii) a custom in-house library compiled of previously identified orchid semiochemicals and synthetic analogues and intermediates. Where synthetic standards were available, co-injections of candidate compounds were conducted on two columns (column 1: VF5-MS column (30 m × 0.25 mm × 0.25 μm film thickness, Varian, Palo Alto, CA, USA), column 2: ATWAX MS column (30 m × 0.25 mm × 0.25 μm film thickness, Grace Discovery Sciences, Columbia, MD, USA)) to confirm their identities.

#### 4.7.2. Screening of Floral Extracts for Electrophysiologically Active Compounds

To determine which populations of flowers contained which electrophysiologically active compounds, we screened for the presence of electrophysiologically active compounds in 347 single flower extracts from the 28 populations for which pollinator response data were collected in the present or previous studies. The compounds that were selected for screening had either been detected previously [42,46], or were found to have an EAD response for *Z. dilatatus* in the present study. Floral extractions and GC/MS were conducted as described previously above, though the pre-extraction baiting step was not included. The mass spectra of electrophysiologically active compounds were added to an AMDIS [80] target library, which was used to individually screen each extract. Mass spectra and retention times were manually checked when a library hit occurred. The default AMDIS search settings were used with the exception of ‘Sensitivity’, which was set to ‘High’.

### 4.8. Compounds That Differ between Ecotypes

To determine whether the attraction of different pollinator species was correlated with the presence of specific compounds, independent of electrophysiological activity, a candidate subset of informative floral compounds was compiled. This subset comprised compounds found to be associated with the attraction of one or two, but not all three, pollinator species.

To detect compounds consistently present in one or more putative ecotypes, but absent from others, the 347 single labellum extracts used in the screening for electrophysiologically active compounds were further analysed. Peak detection and deconvolution was conducted using the EasyGC python pipeline (https://libraries.io/github/dkainer/easyGC, accessed on 13 January 2022), based on PyMS python library [75]) with the default parameters. Data were analysed qualitatively by assessing differences in the suite of compounds present in flowers. Data were presence-absence transformed, with all values greater than zero being set to one, prior to analyses. Compounds were sorted according to the pollinator species of the orchid population using the R packages ‘data.table’ [81] and ‘reshape’ [82]. Compounds present in flowers of all three putative ecotypes were removed, leaving only compounds associated with the attraction of one or two pollinator species. These candidate informative compounds were manually checked using AMDIS (version 2.5) and any peaks determined incorrectly by the software were removed. Candidate compounds were identified using the protocol previously described for electrophysiologically active compounds.

Retention indices and mass spectra of the candidate compounds were compared to those of matches returned by searching: (i) the National Institute of Standards and Technology (NIST-17) database, and (ii) a custom in-house library compiled of previously identified orchid semiochemicals and synthetic analogues and intermediates. Where synthetic standards were available, co-injections of candidate compounds were conducted on two columns as described for compounds detected in the electroantennographic experiments.

## Figures and Tables

**Table 1:** Number of wasps of each species of pollinator recorded at populations that attract different pollinator species. Bold rows indicate the local populations that attract the responding pollinator species. * denotes differences in the number of pollinators observed at populations that attract different pollinator species *p* < 0.05.

*Zaspilothynnus nigripes* Responses	*N* Sites Surveyed	% Sites Present	Average Number of Wasps per Survey ± SE	Total Wasps Observed
***Zaspilothynnus nigripes* pollinated populations**	**9**	**100**	12.00 ± 2.49 *	**108**
*Catocheilus* sp. pollinated populations	12	66.7	4.42 ± 1.58	53
*Zaspilothynnus dilatatus* pollinated populations	7	71.4	3.00 ± 1.11	21
*Zaspilothynnus dilatatus* responses				
*Zaspilothynnus nigripes* pollinated populations	9	0	0	0
*Catocheilus* sp. pollinated populations	12	0	0	0
***Zaspilothynnus dilatatus* pollinated populations**	**7**	**85.7**	2.14 ± 0.46 *	**15**
*Catocheilus* sp. responses				
*Zaspilothynnus nigripes* pollinated populations	9	0	0	0
***Catocheilus* sp. pollinated populations**	**12**	**0**	**0**	**0**
*Zaspilothynnus dilatatus* pollinated populations	not surveyed	not surveyed	not surveyed	not surveyed

**Table 2:** Characteristic mass fragments and retention indices (RI) of informative compounds detected by gas chromatography/mass spectrometry-electroantennographic detection and extract analysis.

No.	Pollinator Association	Name	Characteristic Mass Fragments (EI)	RI	Detection Method
**1**	*Zaspilothynnus nigripes*	2-hydroxymethyl- 3-(3-methylbutyl)- 5-methylpyrazine	194, 163, 138, 109	1532	EAD [46]/Extract analyses
**2**	*Zaspilothynnus nigripes*	Unknown 1	168, 150, 139, 122	1557	Extract analyses
**3**	*Zaspilothynnus nigripes*	Unknown 2	196, 154, 136, 108	1804	Extract analyses
**4**	*Zaspilothynnus nigripes/Catocheilus* sp.	4-(2-hydroxyethyl)-2-methoxyphenol (homovanillyl alcohol)	168, 150, 137, 122	1547	Extract analyses
**5**	*Zaspilothynnis nigripes/Catocheilus* sp.	Unknown 8	208, 124, 107, 77	1722	Extract analyses
**6**	*Zaspilothynnis nigripes/Z. dilatatus*	Heneicosene (unknown isomer)	294, 11, 97, 83, 55	2086	Extract analyses
**7**	*Catocheilus* sp.	3,5,6-trimethylpyrazine-2-carbaldehyde	150, 122, 121, 107	1207	Extract analyses
**8**	*Catocheilus* sp.	2-hydroxymethyl-3,5,6-trimethylpyrazine	152, 151, 134, 123	1299	EAD [42]/Extract analyses
**9**	*Catocheilus* sp.	2-(3-methylbutyl)-3,5,6-trimethylpyrazine	191, 177, 149, 136	1389	EAD [42]/Extract analyses
**10**	*Catocheilus* sp.	Unknown 3	168, 151, 139, 121	1538	Extract analyses
**11**	*Catocheilus* sp.	Unknown 4	208, 193, 175, 149	1568	Extract analyses
**12**	*Catocheilus* sp.	(3,6-dimethylpyrazin-2-yl)methyl 3-methylbutanoate	222, 180, 138, 121	1580	EAD [42]/Extract analyses
**13**	*Catocheilus* sp.	(3,5,6-trimethylpyrazin-2-yl)methyl-3-methylbutanoate	236, 208, 152, 151	1660	EAD [42]/Extract analyses
**14**	*Catocheilus* sp.	(3,5,6-trimethylpyrazin-2-yl)methyl(2*S*)-methylbutanoate	236, 194, 152, 151	1667	EAD [42]/Extract analyses
**15**	*Catocheilus* sp.	Unknown 5	252, 168, 151, 138	1899	Extract analyses
**16**	*Catocheilus* sp.	Unknown 7	253, 168, 151, 121	2001	Extract analyses
**17**	*Catocheilus* sp.	Unknown 6	210, 168, 151, 122	2022	Extract analyses
**18**	*Zaspilothynnus dilatatus*	2-(methylthio)benzene-1,4-diol	156, 141, 113, 97	1507	EAD
**19**	*Zaspilothynnus dilatatus*	4-hydroxy-3-(methylthio)benzaldehyde	168, 167, 139, 97	1507	EAD/Extract analyses
**20**	*Zaspilothynnus dilatatus*	4-(hydroxymethyl)-2-(methylthio)phenol	170, 153, 141, 123	1560	Extract analyses

## Supplementary Materials

The following supporting information can be downloaded at: https://www.mdpi.com/article/10.3390/plants11030260/s1, Figure S1: Average number and behaviour of *Zaspilothynnus nigripes* responding to flowers from populations attracting *Z. nigripes* and populations attracting *Z. dilatatus* at Ruabon Nature Reserve per trial. Error bars denote standard error. Each wasp is included in one category only—the approach category includes only wasps that approached but did not land nor flip the hinge, the land cateogry includes only wasps that approached and landed but did not flip the hinge, and the hinge flip category includes only wasps that approached, landed, and flipped the hinge. Figure S2: Axes two and three of principle coordinate analyses based on the (A) quantitative and (B) presence-absence data from the 66 compounds detected in the Drakaea livida extracts (flowers that attracted *Zaspilothynnus nigripes* = yellow, flowers from populations attracting *Catocheilus* sp. = blue, flowers that attracted *Zaspilothynnus dilatatus* = red). The relative corrected Eigen values denoting the percentage contribution of each axis to the total variation is displayed in the axes titles. Figure S3: (a) Total ion count of synthetic-spiked extract of flowers from populations attracting *Zaspilothynnuz dilatatus* with ion 168 (indicating the presence of 4- hydroxy-3-(methylthio)benzaldehyde) shown in red, and ion 156 (indicating the presence of 2-(methylthio)benzene-1,4-diol) shown in blue, with (b) two responses from different *Z. dilatatus* antennae beneath. Figure S4: Full mass spectra of 1–20. Table S1: Species and behaviour of wasps attracted to flowers from different populations of *Drakaea livida* and the number of flowers of each population that were baited with. Table S2: Voucher specimens numbers, ecotypes, and locations of populations of *Drakaea livida* included in the present study.
