# Supplementary material for: Three Chemically Distinct Floral Ecotypes in Drakaea livida, an Orchid Pollinated by Sexual Deception of Thynnine Wasps
Source: Plants (Basel). 2022 Jan 19;11(3):260. doi: 10.3390/plants11030260 (PMC8840651; doi:10.3390/plants11030260)
Supplement: Supplementary file 1 [file plants-11-00260-s001.zip › plants-1521991-SI.pdf]

SUPPLEMENTARY INFORMATION

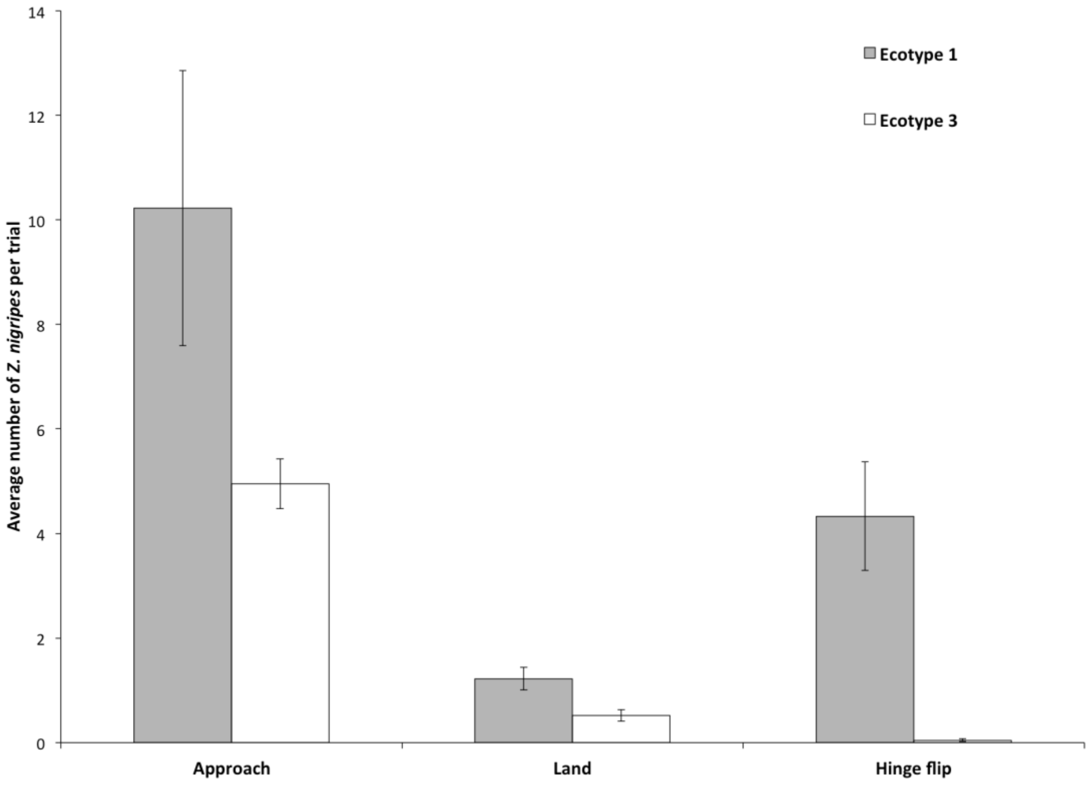

Supplementary Figure S1: Average number and behaviour of *Zaspilothynnus nigripes* responding to flowers from populations attracting *Z. nigripes* and populations attracting *Z. dilatatus* at Ruabon Nature Reserve per trial. Error bars denote standard error. Each wasp is included in one category only - the approach category includes only wasps that approached but did not land nor flip the hinge, the land cateogry includes only wasps that approached and landed but did not flip the hinge, and the hinge flip category includes only wasps that approached, landed, and flipped the hinge.

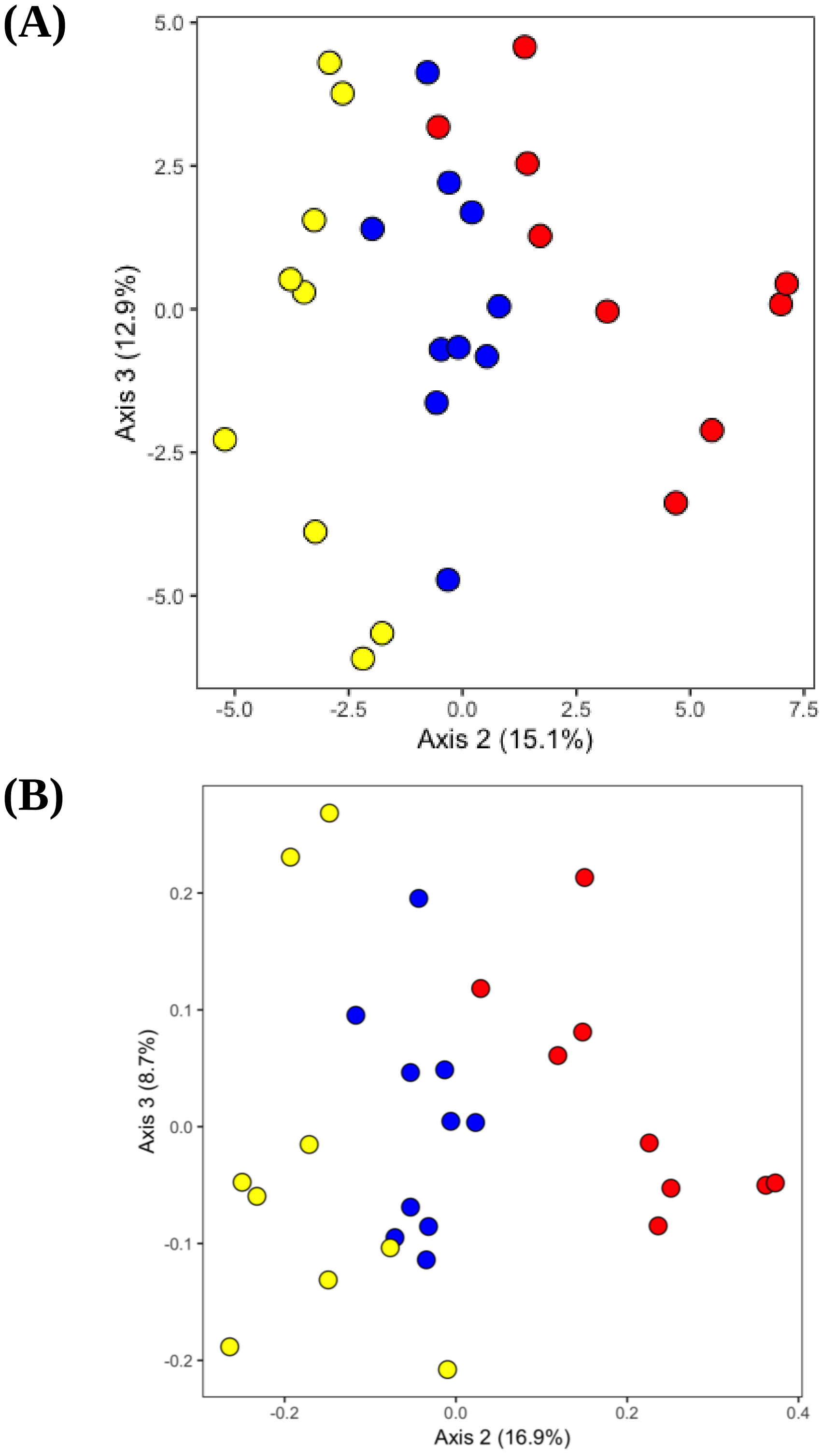

Supplementary Figure S2: Axes two and three of principle coordinate analyses based on the (A) quantitative and (B) presence-absence data from the 66 compounds detected in the *Drakaea livida* extracts (flowers that attracted *Zaspilothynnus nigripes* = yellow, flowers from populations attracting *Catocheilus* sp. = blue, flowers that attracted *Zaspilothynnus dilatatus* = red). The relative corrected Eigen values denoting the percentage contribution of each axis to the total variation is displayed in the axes titles.

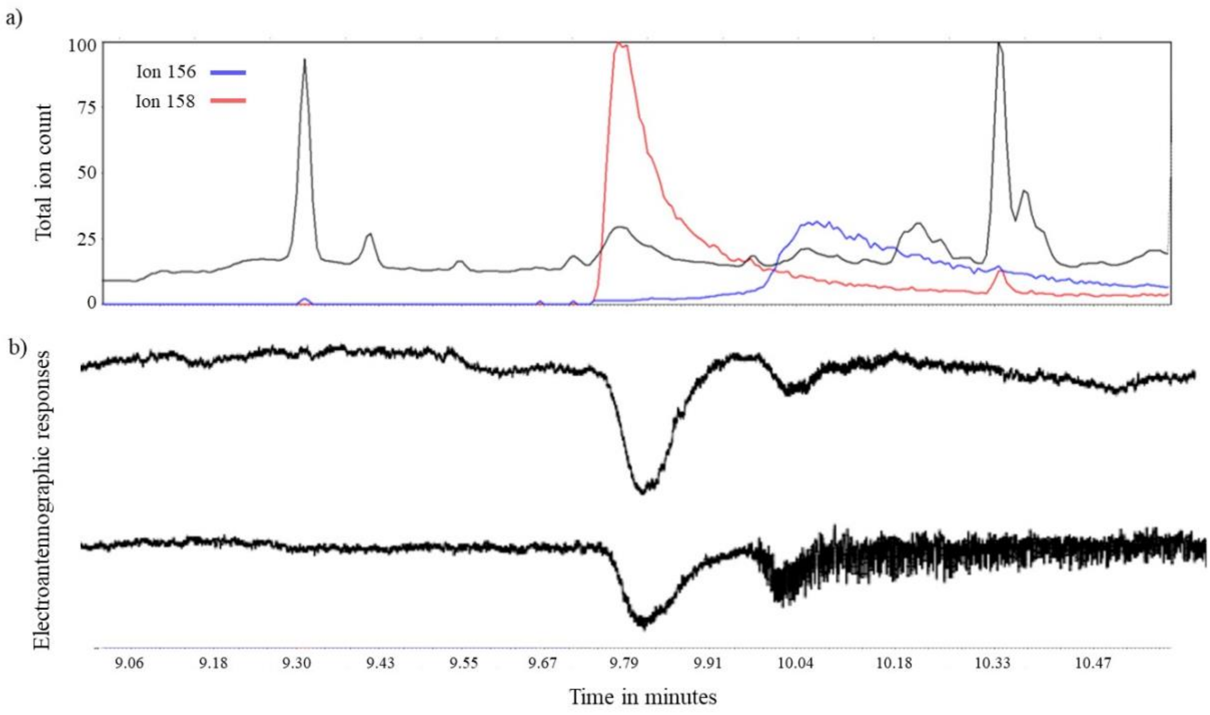

Supplementary Figure S3: (a) Total ion count of synthetic-spiked extract of flowers from populations attracting *Zaspilothynnuz dilatatus* with ion 168 (indicating the presence of 4-hydroxy-3-(methylthio)benzaldehyde) shown in red, and ion 156 (indicating the presence of 2-(methylthio)benzene-1,4-diol) shown in blue, with (b) two responses from different Z. dilatatus antennae beneath.

Supplementary Figure S4

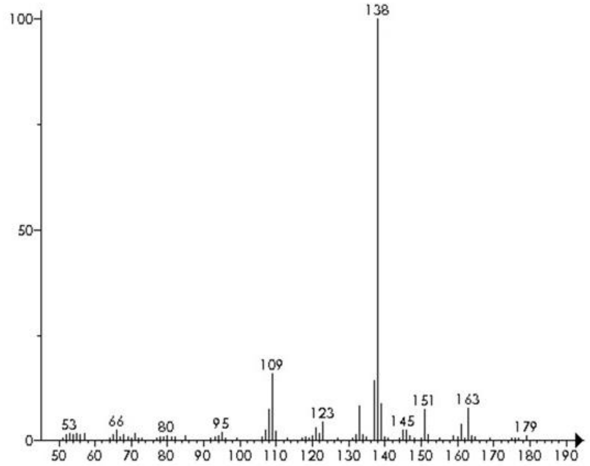

Hydroxymethyl- 3-(3-methylbutyl)- 5-methylpyrazine (RI 1532)

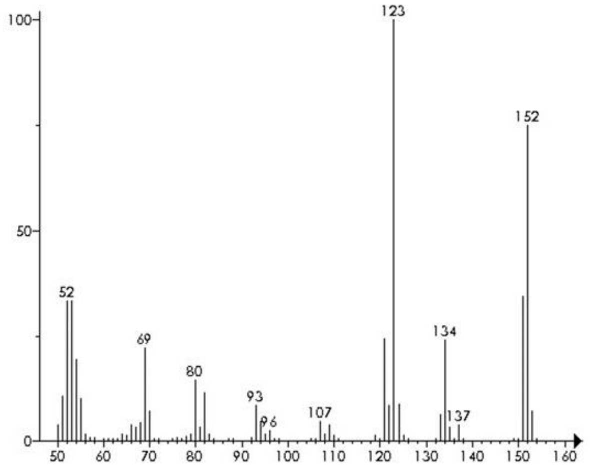

2-hydroxymethyl-3,5,6-trimethylpyrazine (RI 1299)

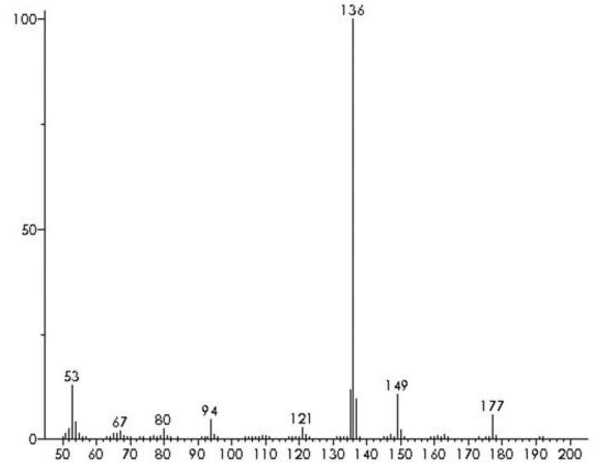

2-(3-methylbutyl)-3,5,6-trimethylpyrazine (RI 1389)

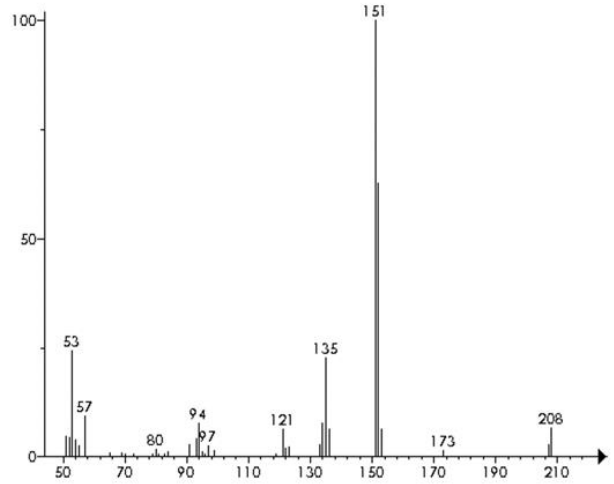

(3,5,6-trimethylpyrazin-2- yl)methyl-3-methylbutanoate (RI 1660)

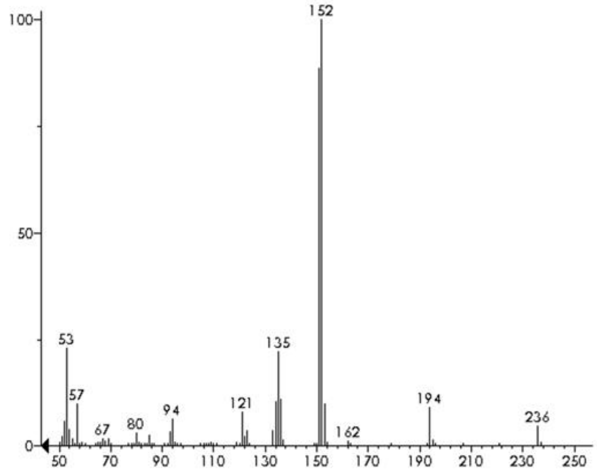

(3,5,6-trimethylpyrazin-2-yl)methyl(2S)-methylbutanoate (RI 1667)

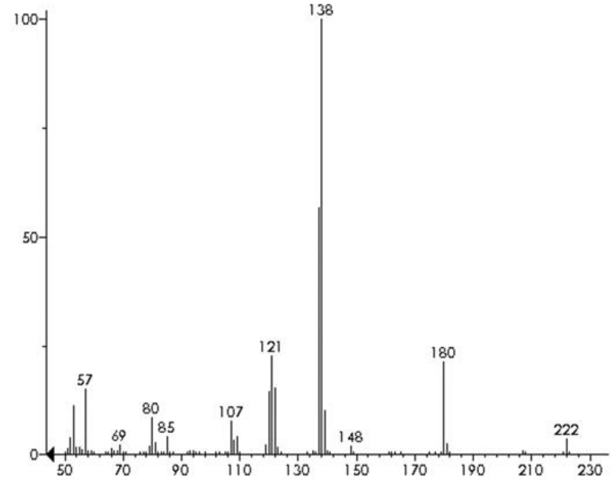

(3,6-dimethylpyrazin-2-yl)methyl 3-methylbutanoate (RI 1580)

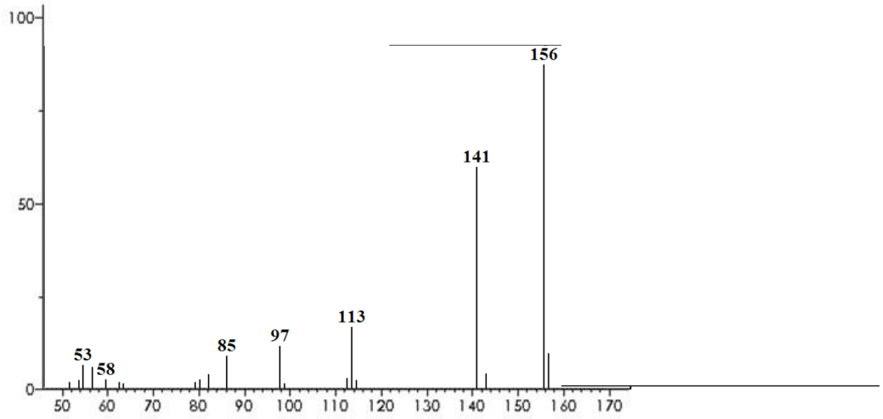

2-(methylthio)benzene-1,4-diol (RI 1507)

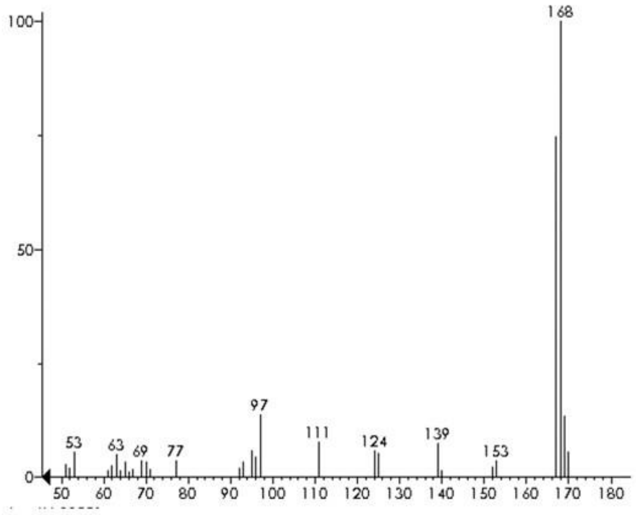

4-hydroxy-3-(methylthio)benzaldehyde (RI 1507)

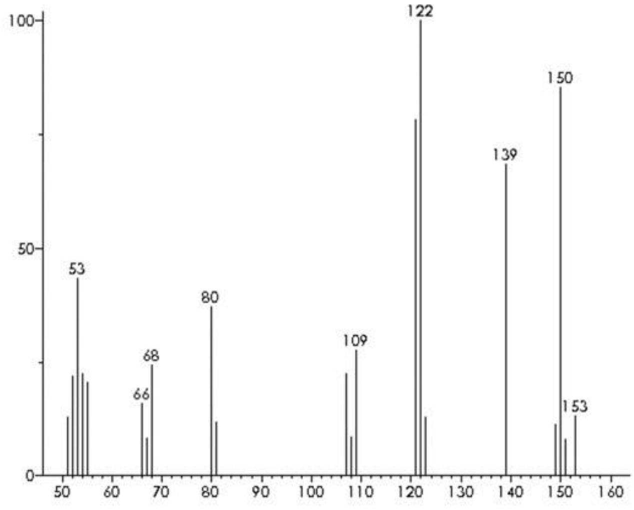

Unknown 1 (RI 1557)

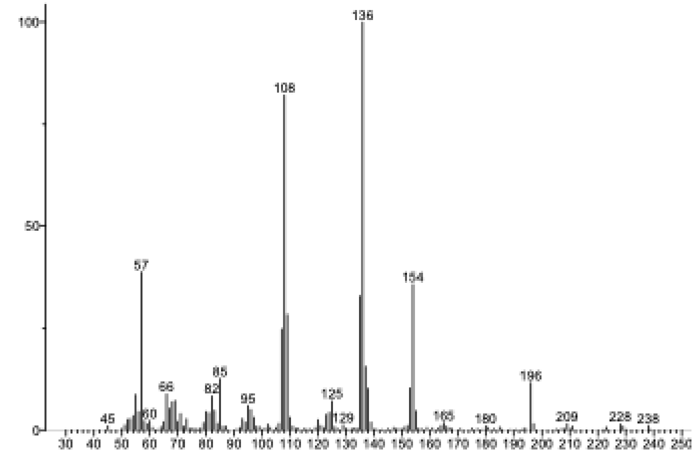

Unknown 2 (RI 1804)

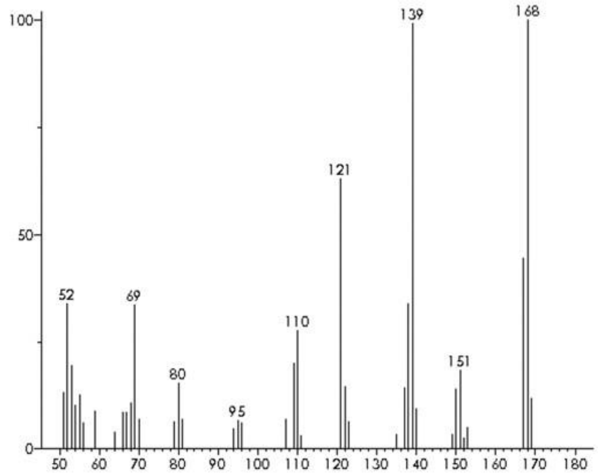

Unknown 3 (RI 1538)

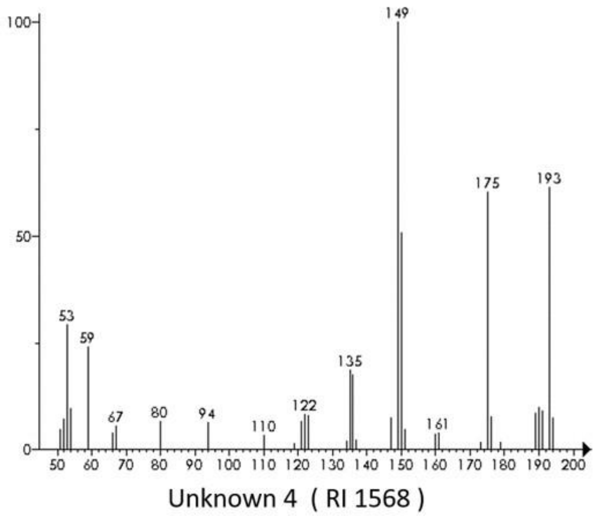

Unknown 4 (RI 1568)

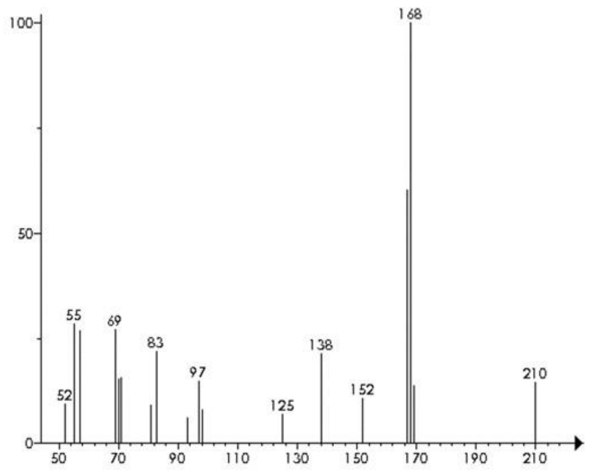

Unknown 5 (RI 1899)

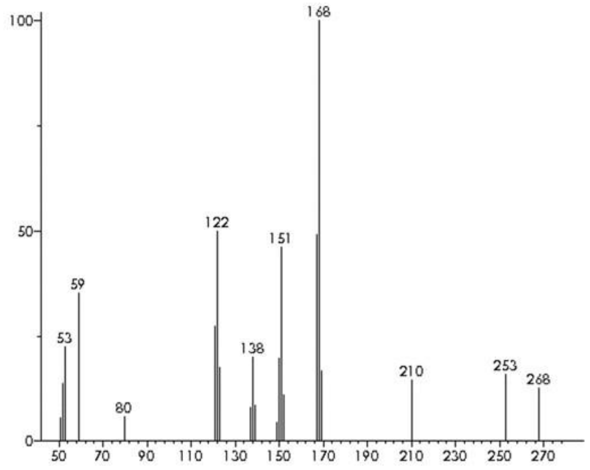

Unknown 6 (RI 2022)

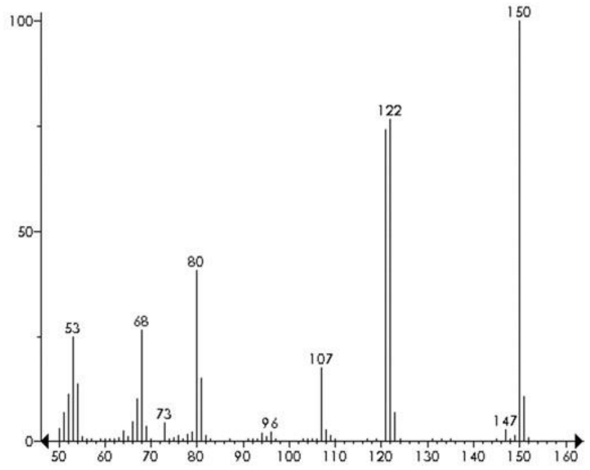

3,5,6-trimethylpyrazine-2-carbaldehyde (RI 1207)

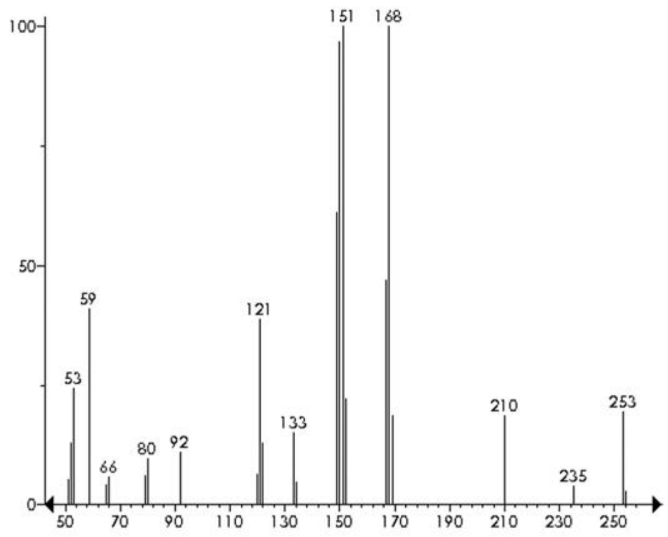

Unknown 7 (RI 2001)

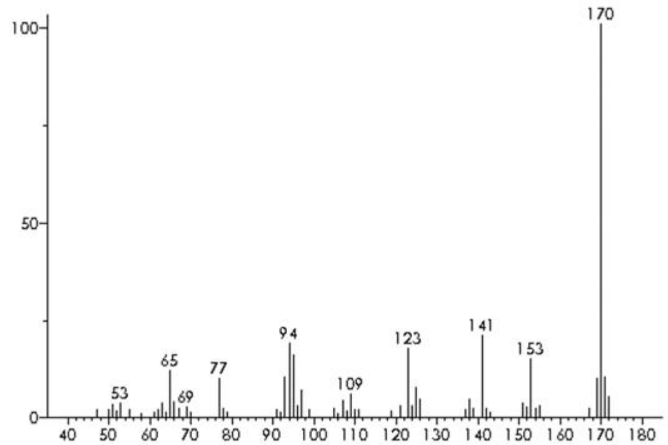

4-(hydroxymethyl)-2-(methylthio)phenol (RI 1560)

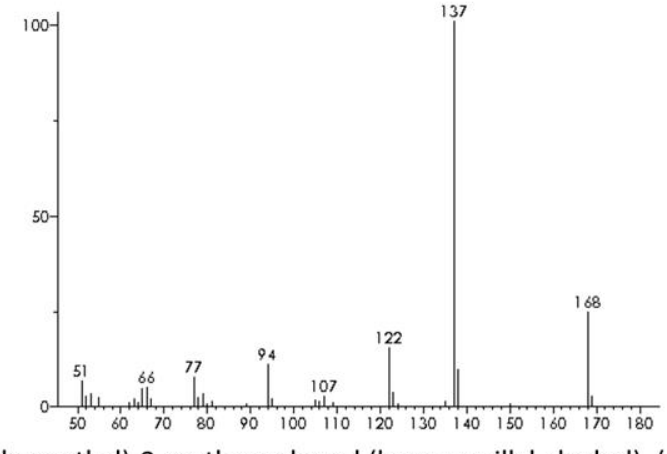

4-(2-hydroxyethyl)-2-methoxyphenol (homovanillyl alcohol) (RI 1547)

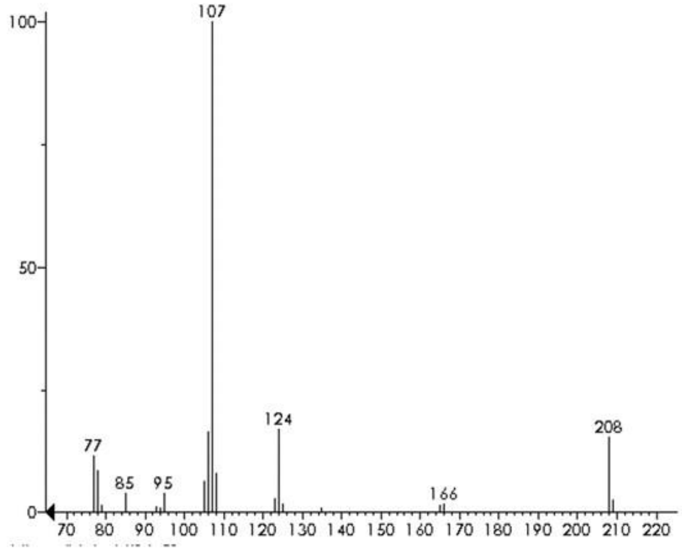

Unknown 8 (RI 1722)

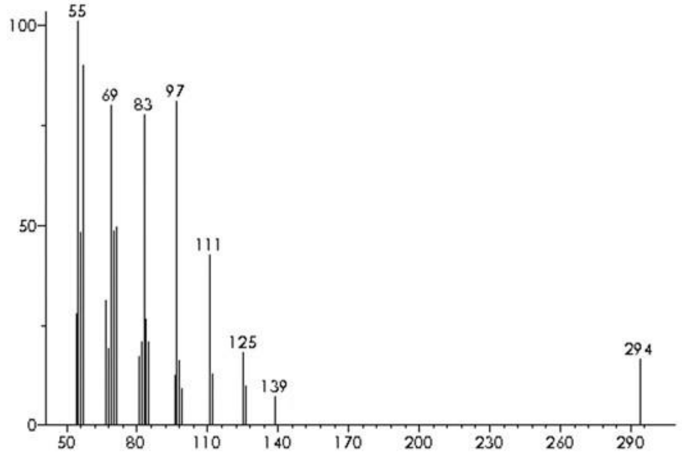

10-heneicosene (Tentatively identified RI 2086)

Supplementary Table S1: Species and behaviour of wasps attracted to flowers from different populations of *Drakaea livida* and the number of flowers of each population that were baited with.

**Population**	**Species caught**	**Wasp behaviour observed**	**No. of flowers baited with**	**IBRA Subregion**
Albany King River	*Zaspilothynnus nigripes*	Hinge flip	1	Southern Jarrah Forest
Bayonet Head	*Zaspilothynnus nigripes*	Hinge flip	1	Southern Jarrah Forest
Blue Lake Road Clearing	*Zaspilothynnus nigripes*	Hinge flip	4	Southern Jarrah Forest
Chesapeake track	*Zaspilothynnus nigripes*	Hinge flip	1	Warren
Granite Road	*Zaspilothynnus nigripes*	Hinge flip	1	Southern Jarrah Forest
Grays Road	*Zaspilothynnus nigripes*	Hinge flip	1	Warren
Isle Road	*Zaspilothynnus nigripes*	Hinge flip	2	Warren
Lane Poole Road	*Zaspilothynnus nigripes*	Hinge flip	5	Warren
Mabinup Track	*Zaspilothynnus nigripes*	Hinge flip	1	Esperance Plains
Mount Lindsey	*Zaspilothynnus nigripes*	Hinge flip	1	Southern Jarrah Forest
Northcliffe Outcrop	*Zaspilothynnus nigripes*	Hinge flip	1	Warren
Qualen Road	*Zaspilothynnus nigripes*	Hinge flip	1	Northern Jarrah Forest
Rainbow Cave Road	*Zaspilothynnus nigripes*	Hinge flip	7	Warren
Scotsdale Outcrop	*Zaspilothynnus nigripes*	Hinge flip	1	Warren
Spencer Road	*Zaspilothynnus nigripes*	Hinge flip	20	Warren
SW Hwy Outcrop	*Zaspilothynnus nigripes*	Hinge flip	2	Warren
Blue Lake Road	*Catocheilus* sp.	Close approach (<5cm)	3	Southern Jarrah Forest
Blue Lake Road Sand Patch	*Catocheilus* sp.	Close approach (<5cm)	1	Southern Jarrah Forest
Frosty Road	*Catocheilus* sp.	Close approach (<5cm)	9	Southern Jarrah Forest
Greenbushes	*Catocheilus* sp.	Close approach (<5cm)	2	Southern Jarrah Forest
Mowen Road	*Catocheilus* sp.	Close approach (<5cm)	2	Southern Jarrah Forest
Carrabungup Nature Reserve	*Zaspilothynnus dilatatus*	Hinge flip	5	Swan Coastal Plain
Franklandia Nature Reserve	*Zaspilothynnus dilatatus*	Hinge flip	3	Swan Coastal Plain
Goodale Sanctuary	*Zaspilothynnus dilatatus*	Hinge flip	5	Swan Coastal Plain
Island Point Nature Reserve	*Zaspilothynnus dilatatus*	Hinge flip	14	Swan Coastal Plain
Johnston Road	*Zaspilothynnus dilatatus*	Hinge flip	1	Swan Coastal Plain
Manea Park	*Zaspilothynnus dilatatus*	Hinge flip	3	Swan Coastal Plain
Serpentine River Nature Reserve	*Zaspilothynnus dilatatus*	Hinge flip	12	Swan Coastal Plain

Supplementary Table S2: Voucher specimens numbers, ecotypes, and locations of populations of *Drakaea livida* included in the present study

Population Location	Ecotype	Voucher Number	Latitude & Longitude
Albany King River	Ecotype One	PERTH 09005633	-34.933°, 117.891°
Bayonet Head	Ecotype One	PERTH 09005579	-34.974°, 117.937°
Lane Poole Northcliffe	Ecotype One	PERTH 09005765	-34.577°, 116.197°
Mount Lindsey	Ecotype One	PERTH 09005757	-34.854°, 117.241°
Northcliffe Outcrop	Ecotype One	PERTH 09005730	-34.778°, 116.081°
Qualen Road	Ecotype One	PERTH 05493714	-32.108°, 116.652°
Rainbow Cave Road	Ecotype One	PERTH 08605254	-34.004°, 115.022°
Scotsdale Outcrop	Ecotype One	PERTH 09005587	-34.850°, 117.261°
Spencer Road	Ecotype One	PERTH 09005773	-33.706°, 115.027°
SW Hwy Outrcrop	Ecotype One	PERTH 08604584	-34.758°, 116.501°
Isle Road	Ecotype One	PERTH 08603561	-34.988°, 116.695°
Granite Road	Ecotype One	PERTH 09005749	-34.840°, 117.251°
Frosty Road	Ecotype Two	PERTH 09005609	-34.357°, 116.385°
Mowen Road	Ecotype Two	PERTH 09005714	-33.924°, 115.396°
Carrabungup Nature Reserve	Ecotype Three	PERTH 09048014	-32.647°, 115.715°
Franklandia Nature Reserve	Ecotype Three	PERTH 09005692	-33.425°, 115.697°
Goodale Sanctuary	Ecotype Three	PERTH 09005668	-32.722°, 115.775°
Island Point Nature Reserve	Ecotype Three	PERTH 09005706	-32.757°, 115.690°
Manea Park	Ecotype Three	PERTH 09005595	-33.382°, 115.657°
Serpentine River Nature Reserve	Ecotype Three	PERTH 08739889	-32.335°, 115.791°
